# 4-(2,3-Dimeth­oxy­phen­yl)-1*H*-pyrrole-3-carbonitrile

**DOI:** 10.1107/S1600536812018302

**Published:** 2012-04-28

**Authors:** Qing-Hao Chen, Fan-Wei Meng, Gui-Jun Dong, Xiao-Dan Wang, Jin-Sheng Gao

**Affiliations:** a Engineering Research Center of Pesticides of Heilongjiang University, Heilongjiang University, Harbin 150050, People’s Republic of China; b Dalian Songliao Chemical Industry Corporation, Dalian 116031, People’s Republic of China

## Abstract

The asymmetric unit of the title compound, C_13_H_12_N_2_O_2_, obtained in a search for analogs of the fungicide fludioxonil [systematic name: 4-(2,2-difluoro-1,3-benzodioxol-4-yl)-1*H*-pyrrole-3-carbonitrile], contains two independent mol­ecules, *A* and *B*. The benzene and pyrrole rings are inclined to each other at 38.5 (1) and 29.3 (1)° in mol­ecules *A* and *B*, respectively. In the crystal, bifurcated N—H⋯(O,O) hydrogen bonds link *A* mol­ecules into chains along [001], while *B* mol­ecules are linked into layers parallel to the *bc* plane *via* bifurcated N—H⋯(N,N) hydrogen bonds.

## Related literature

For the synthesis of the title compound, see: Pfluger *et al.* (1989 ▶).
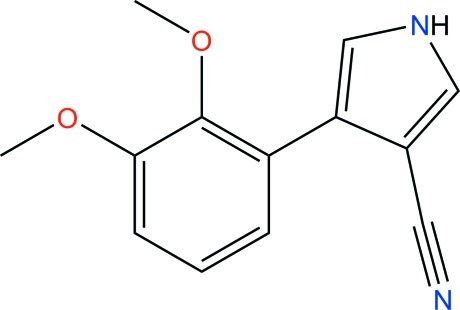


## Experimental

### 

#### Crystal data

C_13_H_12_N_2_O_2_
*M* *_r_* = 228.25
Monoclinic, 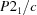

*a* = 17.527 (4) Å
*b* = 9.6576 (19) Å
*c* = 14.237 (3) Å
β = 106.92 (3)°
*V* = 2305.5 (8) Å^3^
*Z* = 8
Mo *K*α radiation
μ = 0.09 mm^−1^
*T* = 293 K
0.69 × 0.67 × 0.53 mm

#### Data collection

Rigaku R-AXIS RAPID diffractometer
Absorption correction: multi-scan (*ABSCOR*; Higashi, 1995 ▶) *T*_min_ = 0.941, *T*_max_ = 0.954
21190 measured reflections
5215 independent reflections
3907 reflections with *I* > 2σ(*I*)
*R*_int_ = 0.042

#### Refinement

*R*[*F* ^2^ > 2σ(*F* ^2^)] = 0.045
*wR*(*F* ^2^) = 0.129
*S* = 1.07
5215 reflections
320 parameters
2 restraints
H atoms treated by a mixture of independent and constrained refinement
Δρ_max_ = 0.36 e Å^−3^
Δρ_min_ = −0.15 e Å^−3^

### 

Data collection: *RAPID-AUTO* (Rigaku, 1998 ▶); cell refinement: *RAPID-AUTO*; data reduction: *CrystalClear* (Rigaku/MSC, 2002 ▶); program(s) used to solve structure: *SHELXS97* (Sheldrick, 2008 ▶); program(s) used to refine structure: *SHELXL97* (Sheldrick, 2008 ▶); molecular graphics: *SHELXTL* (Sheldrick, 2008 ▶); software used to prepare material for publication: *SHELXL97*.

## Supplementary Material

Crystal structure: contains datablock(s) I, global. DOI: 10.1107/S1600536812018302/cv5288sup1.cif

Structure factors: contains datablock(s) I. DOI: 10.1107/S1600536812018302/cv5288Isup2.hkl

Supplementary material file. DOI: 10.1107/S1600536812018302/cv5288Isup3.cml

Additional supplementary materials:  crystallographic information; 3D view; checkCIF report

## Figures and Tables

**Table 1:** Hydrogen-bond geometry (Å, °)

*D*—H⋯*A*	*D*—H	H⋯*A*	*D*⋯*A*	*D*—H⋯*A*
N1—H11⋯N2^i^	0.90 (1)	2.38 (2)	3.086 (2)	136 (2)
N1—H11⋯N2^ii^	0.90 (1)	2.55 (2)	3.250 (2)	136 (2)
N3—H31⋯O4^iii^	0.89 (1)	2.12 (1)	2.9327 (16)	151 (2)
N3—H31⋯O3^iii^	0.89 (1)	2.38 (2)	3.0709 (18)	134 (2)

Symmetry codes: (i) 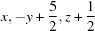; (ii) 
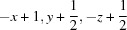
; (iii) 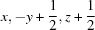.

**Fig. 1.**
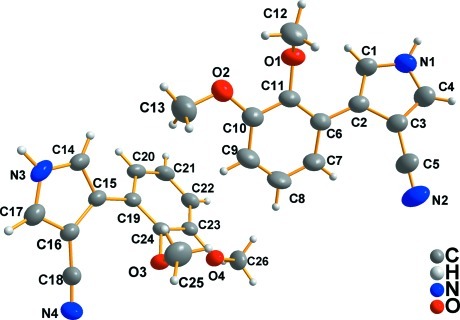
Two independent molecules in the asymmetric unit of (I) showing the atomic numbering and 50% probability displacement ellipsoids.

**Fig. 2.**
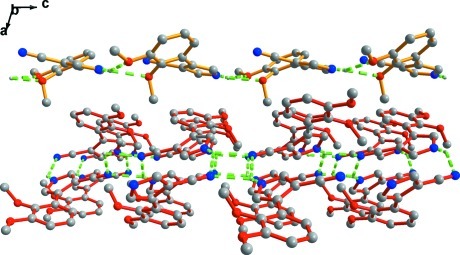
A portion of the crystal packing showing hydrogen bonds as dashed lines. H atoms omitted for clarity.

## supplementary crystallographic information

### Comment

The title compound, (I), is the analogue of Fludioxonil, which is kind of fungicide developed and produced by Novartis. Herein, we report the synthesis and crystal structure of (I).

The asymmetric unit of (I) (Fig. 1), obtained in a search for analogs of Fludioxonil, contains two independent molecules, *A* and *B*, respectively. The benzene and pyrrole rings are inclined to each other at 38.5 (1)° in molecules *A* and 29.3 (1)° in molecules *B*. In the crystal (Fig. 2), intermolecular bifurcated N—H···O hydrogen bonds (Table 1) link molecules *A* into chains in [001], while molecules *B* are linked into layers parallel to *bc* plane *via* bifurcated N—H···N hydrogen bonds (Table 1).

### Experimental

The title compound was prepared by the reaction of (Z)-2-cyano-3-(2,3-dimethoxyphenyl)acrylamide and 1-(isocyanomethylsulfonyl)-4-methylbenzene under alkaline condition (Pfluger *et al.*, 1989). A colourless block crystal suitable for X-ray diffraction was obtained by the recrystallization of (I) from methanol.

### Refinement

H atoms bound to C atoms were placed in calculated positions and treated as riding on their parent atoms, with C—H = 0.93 - 0.96 Å and with *U*_iso_(H) = 1.2 - 1.5 *U*_eq_(C). N-bound H atoms were located in a difference Fourier map and were isotropically refined with restraint O—N = 0.90 (1) Å.

### Crystal data

**Table:**

C_13_H_12_N_2_O_2_	*F*(000) = 960
*M**_r_* = 228.25	*D*_x_ = 1.315 Mg m^−^^3^
Monoclinic, *P*2_1_/*c*	Mo *K*α radiation, λ = 0.71073 Å
Hall symbol: -P 2ybc	Cell parameters from 16853 reflections
*a* = 17.527 (4) Å	θ = 3.0–27.5°
*b* = 9.6576 (19) Å	µ = 0.09 mm^−^^1^
*c* = 14.237 (3) Å	*T* = 293 K
β = 106.92 (3)°	Block, yellow
*V* = 2305.5 (8) Å^3^	0.69 × 0.67 × 0.53 mm
*Z* = 8	

### Data collection

**Table:**

Rigaku R-AXIS RAPID diffractometer	5215 independent reflections
Radiation source: fine-focus sealed tube	3907 reflections with *I* > 2σ(*I*)
Graphite monochromator	*R*_int_ = 0.042
ω scan	θ_max_ = 27.5°, θ_min_ = 3.0°
Absorption correction: multi-scan (*ABSCOR*; Higashi, 1995)	*h* = −22→22
*T*_min_ = 0.941, *T*_max_ = 0.954	*k* = −12→12
21190 measured reflections	*l* = −17→18

### Refinement

**Table:**

Refinement on *F*^2^	Secondary atom site location: difference Fourier map
Least-squares matrix: full	Hydrogen site location: inferred from neighbouring sites
*R*[*F*^2^ > 2σ(*F*^2^)] = 0.045	H atoms treated by a mixture of independent and constrained refinement
*wR*(*F*^2^) = 0.129	*w* = 1/[σ^2^(*F*_o_^2^) + (0.0652*P*)^2^ + 0.2588*P*] where *P* = (*F*_o_^2^ + 2*F*_c_^2^)/3
*S* = 1.07	(Δ/σ)_max_ < 0.001
5215 reflections	Δρ_max_ = 0.36 e Å^−^^3^
320 parameters	Δρ_min_ = −0.15 e Å^−^^3^
2 restraints	Extinction correction: *SHELXL97* (Sheldrick, 2008), Fc^*^=kFc[1+0.001xFc^2^λ^3^/sin(2θ)]^-1/4^
Primary atom site location: structure-invariant direct methods	Extinction coefficient: 0.0100 (14)

### Special details

**Table:**

Geometry. All esds (except the esd in the dihedral angle between two l.s. planes) are estimated using the full covariance matrix. The cell esds are taken into account individually in the estimation of esds in distances, angles and torsion angles; correlations between esds in cell parameters are only used when they are defined by crystal symmetry. An approximate (isotropic) treatment of cell esds is used for estimating esds involving l.s. planes.
Refinement. Refinement of F^2^ against ALL reflections. The weighted R-factor wR and goodness of fit S are based on F^2^, conventional R-factors R are based on F, with F set to zero for negative F^2^. The threshold expression of F^2^ > 2sigma(F^2^) is used only for calculating R-factors(gt) etc. and is not relevant to the choice of reflections for refinement. R-factors based on F^2^ are statistically about twice as large as those based on F, and R- factors based on ALL data will be even larger.

### Fractional atomic coordinates and isotropic or equivalent isotropic displacement parameters (Å^2^)

**Table:**

	*x*	*y*	*z*	*U*_iso_*/*U*_eq_	
C1	0.39205 (11)	1.20321 (18)	0.36418 (13)	0.0544 (4)	
H1	0.3681	1.1873	0.4135	0.065*	
C2	0.38722 (9)	1.11726 (16)	0.28632 (11)	0.0448 (3)	
C3	0.43351 (10)	1.18515 (16)	0.23130 (12)	0.0477 (4)	
C4	0.46329 (11)	1.30598 (18)	0.27889 (13)	0.0568 (4)	
H4	0.4956	1.3695	0.2594	0.068*	
C5	0.45012 (12)	1.13911 (17)	0.14436 (13)	0.0554 (4)	
C6	0.34673 (9)	0.98238 (16)	0.26304 (11)	0.0454 (3)	
C7	0.31916 (11)	0.9373 (2)	0.16570 (12)	0.0570 (4)	
H7	0.3235	0.9955	0.1155	0.068*	
C8	0.28560 (12)	0.8080 (2)	0.14306 (13)	0.0660 (5)	
H8	0.2685	0.7792	0.0779	0.079*	
C9	0.27712 (11)	0.72099 (19)	0.21594 (13)	0.0604 (5)	
H9	0.2545	0.6338	0.1999	0.072*	
C10	0.30215 (10)	0.76306 (17)	0.31290 (12)	0.0506 (4)	
C11	0.33585 (9)	0.89520 (17)	0.33612 (11)	0.0460 (4)	
C12	0.41738 (13)	0.8724 (2)	0.50009 (15)	0.0778 (6)	
H12A	0.4005	0.7807	0.5108	0.117*	
H12B	0.4319	0.9224	0.5610	0.117*	
H12C	0.4626	0.8669	0.4750	0.117*	
C13	0.26965 (15)	0.54613 (19)	0.37083 (17)	0.0765 (6)	
H13A	0.2168	0.5441	0.3259	0.115*	
H13B	0.2696	0.5015	0.4310	0.115*	
H13C	0.3056	0.4985	0.3424	0.115*	
C14	0.14006 (10)	0.23868 (16)	0.28910 (11)	0.0449 (3)	
H14	0.1442	0.3042	0.3382	0.054*	
C15	0.12366 (8)	0.26671 (14)	0.19105 (10)	0.0367 (3)	
C16	0.12262 (9)	0.13441 (14)	0.14436 (11)	0.0401 (3)	
C17	0.13847 (10)	0.03501 (16)	0.21709 (12)	0.0495 (4)	
H17	0.1411	−0.0600	0.2078	0.059*	
C18	0.10455 (10)	0.10123 (13)	0.04287 (12)	0.0463 (4)	
C19	0.10576 (8)	0.40571 (13)	0.14708 (9)	0.0342 (3)	
C20	0.06180 (9)	0.49956 (15)	0.18607 (10)	0.0412 (3)	
H20	0.0452	0.4741	0.2400	0.049*	
C21	0.04304 (9)	0.62910 (15)	0.14515 (11)	0.0444 (3)	
H21	0.0139	0.6897	0.1720	0.053*	
C22	0.06663 (9)	0.67063 (14)	0.06522 (11)	0.0410 (3)	
H22	0.0530	0.7579	0.0379	0.049*	
C23	0.11099 (8)	0.58046 (13)	0.02615 (9)	0.0338 (3)	
C24	0.13089 (8)	0.44940 (12)	0.06761 (9)	0.0321 (3)	
C25	0.25651 (11)	0.3560 (2)	0.07426 (16)	0.0661 (5)	
H25A	0.2804	0.4447	0.0713	0.099*	
H25B	0.2812	0.2877	0.0436	0.099*	
H25C	0.2640	0.3310	0.1416	0.099*	
C26	0.11184 (11)	0.73558 (14)	−0.10410 (12)	0.0512 (4)	
H26A	0.0546	0.7355	−0.1274	0.077*	
H26B	0.1331	0.7424	−0.1589	0.077*	
H26C	0.1296	0.8132	−0.0611	0.077*	
N1	0.43741 (10)	1.31580 (15)	0.35841 (11)	0.0587 (4)	
H11	0.4537 (12)	1.3808 (17)	0.4048 (12)	0.076 (6)*	
N2	0.46384 (13)	1.10171 (18)	0.07500 (13)	0.0791 (5)	
N3	0.14938 (9)	0.09944 (14)	0.30346 (10)	0.0520 (3)	
H31	0.1571 (12)	0.0577 (18)	0.3613 (9)	0.067 (6)*	
N4	0.08913 (12)	0.06891 (14)	−0.03785 (11)	0.0673 (5)	
O1	0.35427 (8)	0.94207 (13)	0.43141 (8)	0.0585 (3)	
O2	0.29473 (8)	0.68584 (13)	0.39028 (9)	0.0643 (3)	
O3	0.17371 (6)	0.36250 (9)	0.02440 (7)	0.0399 (2)	
O4	0.13875 (7)	0.61031 (9)	−0.05192 (7)	0.0430 (3)	

### Atomic displacement parameters (Å^2^)

**Table:**

	*U*^11^	*U*^22^	*U*^33^	*U*^12^	*U*^13^	*U*^23^
C1	0.0560 (10)	0.0636 (10)	0.0478 (9)	−0.0014 (7)	0.0218 (8)	−0.0090 (7)
C2	0.0403 (8)	0.0564 (9)	0.0368 (8)	0.0044 (6)	0.0099 (6)	−0.0027 (6)
C3	0.0506 (9)	0.0526 (8)	0.0402 (8)	0.0041 (7)	0.0139 (7)	0.0015 (6)
C4	0.0611 (11)	0.0568 (9)	0.0547 (10)	−0.0027 (7)	0.0202 (8)	−0.0005 (8)
C5	0.0730 (12)	0.0506 (9)	0.0484 (10)	0.0023 (7)	0.0267 (9)	0.0056 (7)
C6	0.0373 (8)	0.0590 (9)	0.0381 (8)	0.0022 (6)	0.0078 (6)	−0.0060 (7)
C7	0.0553 (10)	0.0752 (11)	0.0369 (9)	−0.0056 (8)	0.0080 (7)	−0.0048 (8)
C8	0.0687 (12)	0.0823 (13)	0.0395 (9)	−0.0126 (9)	0.0041 (8)	−0.0158 (9)
C9	0.0592 (11)	0.0633 (10)	0.0512 (10)	−0.0085 (8)	0.0043 (8)	−0.0137 (8)
C10	0.0459 (9)	0.0583 (9)	0.0445 (9)	−0.0010 (7)	0.0082 (7)	−0.0042 (7)
C11	0.0393 (8)	0.0597 (9)	0.0364 (8)	0.0012 (6)	0.0070 (6)	−0.0083 (7)
C12	0.0730 (14)	0.0918 (15)	0.0520 (12)	−0.0152 (11)	−0.0078 (10)	0.0013 (10)
C13	0.0929 (16)	0.0524 (10)	0.0781 (14)	−0.0012 (10)	0.0154 (12)	0.0024 (9)
C14	0.0548 (9)	0.0509 (8)	0.0335 (8)	−0.0055 (6)	0.0198 (7)	0.0001 (6)
C15	0.0418 (7)	0.0404 (7)	0.0316 (7)	−0.0012 (5)	0.0163 (6)	−0.0001 (5)
C16	0.0520 (9)	0.0369 (7)	0.0359 (7)	0.0040 (6)	0.0201 (6)	0.0031 (5)
C17	0.0647 (10)	0.0432 (8)	0.0461 (9)	0.0056 (7)	0.0251 (8)	0.0094 (6)
C18	0.0721 (11)	0.0280 (6)	0.0448 (9)	0.0042 (6)	0.0263 (8)	0.0019 (6)
C19	0.0388 (7)	0.0358 (6)	0.0278 (6)	−0.0021 (5)	0.0095 (5)	−0.0047 (5)
C20	0.0435 (8)	0.0484 (8)	0.0353 (7)	−0.0013 (6)	0.0169 (6)	−0.0097 (6)
C21	0.0433 (8)	0.0454 (8)	0.0457 (8)	0.0069 (6)	0.0147 (7)	−0.0145 (6)
C22	0.0461 (8)	0.0329 (6)	0.0411 (8)	0.0054 (5)	0.0082 (6)	−0.0064 (6)
C23	0.0404 (7)	0.0315 (6)	0.0278 (6)	−0.0012 (5)	0.0070 (5)	−0.0049 (5)
C24	0.0377 (7)	0.0306 (6)	0.0284 (6)	0.0005 (5)	0.0105 (5)	−0.0067 (5)
C25	0.0521 (11)	0.0758 (12)	0.0785 (14)	0.0151 (8)	0.0316 (10)	−0.0034 (10)
C26	0.0683 (11)	0.0337 (7)	0.0520 (9)	0.0018 (6)	0.0180 (8)	0.0101 (6)
N1	0.0666 (10)	0.0571 (8)	0.0538 (9)	−0.0045 (7)	0.0197 (7)	−0.0142 (7)
N2	0.1202 (16)	0.0723 (10)	0.0624 (11)	−0.0016 (10)	0.0540 (11)	0.0017 (8)
N3	0.0651 (9)	0.0563 (8)	0.0386 (7)	0.0013 (6)	0.0215 (6)	0.0155 (6)
N4	0.1232 (15)	0.0392 (7)	0.0459 (9)	−0.0045 (8)	0.0349 (9)	−0.0068 (6)
O1	0.0641 (8)	0.0702 (7)	0.0402 (6)	−0.0024 (6)	0.0139 (5)	−0.0099 (5)
O2	0.0721 (9)	0.0633 (7)	0.0540 (7)	−0.0162 (6)	0.0129 (6)	−0.0030 (6)
O3	0.0560 (6)	0.0333 (5)	0.0364 (5)	0.0080 (4)	0.0229 (5)	−0.0015 (4)
O4	0.0650 (7)	0.0311 (5)	0.0372 (5)	0.0047 (4)	0.0214 (5)	0.0041 (4)

### Geometric parameters (Å, º)

**Table:**

C1—N1	1.363 (2)	C14—C15	1.368 (2)
C1—C2	1.368 (2)	C14—H14	0.9300
C1—H1	0.9300	C15—C16	1.4379 (19)
C2—C3	1.439 (2)	C15—C19	1.4758 (19)
C2—C6	1.474 (2)	C16—C17	1.380 (2)
C3—C4	1.374 (2)	C16—C18	1.423 (2)
C3—C5	1.423 (2)	C17—N3	1.341 (2)
C4—N1	1.340 (2)	C17—H17	0.9300
C4—H4	0.9300	C18—N4	1.145 (2)
C5—N2	1.141 (2)	C19—C24	1.3937 (18)
C6—C11	1.394 (2)	C19—C20	1.4044 (18)
C6—C7	1.398 (2)	C20—C21	1.379 (2)
C7—C8	1.378 (3)	C20—H20	0.9300
C7—H7	0.9300	C21—C22	1.379 (2)
C8—C9	1.376 (3)	C21—H21	0.9300
C8—H8	0.9300	C22—C23	1.3871 (18)
C9—C10	1.382 (2)	C22—H22	0.9300
C9—H9	0.9300	C23—O4	1.3674 (16)
C10—O2	1.368 (2)	C23—C24	1.3972 (18)
C10—C11	1.405 (2)	C24—O3	1.3837 (15)
C11—O1	1.3765 (18)	C25—O3	1.419 (2)
C12—O1	1.415 (2)	C25—H25A	0.9600
C12—H12A	0.9600	C25—H25B	0.9600
C12—H12B	0.9600	C25—H25C	0.9600
C12—H12C	0.9600	C26—O4	1.4261 (17)
C13—O2	1.421 (2)	C26—H26A	0.9600
C13—H13A	0.9600	C26—H26B	0.9600
C13—H13B	0.9600	C26—H26C	0.9600
C13—H13C	0.9600	N1—H11	0.896 (9)
C14—N3	1.363 (2)	N3—H31	0.891 (9)
			
N1—C1—C2	109.57 (15)	C16—C15—C19	129.77 (12)
N1—C1—H1	125.2	C17—C16—C18	122.83 (13)
C2—C1—H1	125.2	C17—C16—C15	107.38 (13)
C1—C2—C3	104.65 (14)	C18—C16—C15	129.69 (12)
C1—C2—C6	129.11 (15)	N3—C17—C16	108.00 (14)
C3—C2—C6	126.21 (14)	N3—C17—H17	126.0
C4—C3—C5	123.36 (16)	C16—C17—H17	126.0
C4—C3—C2	108.18 (14)	N4—C18—C16	177.05 (16)
C5—C3—C2	128.43 (15)	C24—C19—C20	117.71 (12)
N1—C4—C3	107.66 (15)	C24—C19—C15	122.78 (11)
N1—C4—H4	126.2	C20—C19—C15	119.52 (12)
C3—C4—H4	126.2	C21—C20—C19	120.62 (13)
N2—C5—C3	179.6 (2)	C21—C20—H20	119.7
C11—C6—C7	117.97 (15)	C19—C20—H20	119.7
C11—C6—C2	121.82 (13)	C22—C21—C20	121.39 (12)
C7—C6—C2	120.20 (15)	C22—C21—H21	119.3
C8—C7—C6	121.00 (17)	C20—C21—H21	119.3
C8—C7—H7	119.5	C21—C22—C23	119.08 (13)
C6—C7—H7	119.5	C21—C22—H22	120.5
C9—C8—C7	120.61 (16)	C23—C22—H22	120.5
C9—C8—H8	119.7	O4—C23—C22	124.55 (12)
C7—C8—H8	119.7	O4—C23—C24	115.48 (11)
C8—C9—C10	120.06 (17)	C22—C23—C24	119.97 (13)
C8—C9—H9	120.0	O3—C24—C19	121.18 (11)
C10—C9—H9	120.0	O3—C24—C23	117.56 (11)
O2—C10—C9	124.55 (16)	C19—C24—C23	121.21 (11)
O2—C10—C11	115.97 (14)	O3—C25—H25A	109.5
C9—C10—C11	119.45 (16)	O3—C25—H25B	109.5
O1—C11—C6	119.56 (14)	H25A—C25—H25B	109.5
O1—C11—C10	119.51 (14)	O3—C25—H25C	109.5
C6—C11—C10	120.84 (14)	H25A—C25—H25C	109.5
O1—C12—H12A	109.5	H25B—C25—H25C	109.5
O1—C12—H12B	109.5	O4—C26—H26A	109.5
H12A—C12—H12B	109.5	O4—C26—H26B	109.5
O1—C12—H12C	109.5	H26A—C26—H26B	109.5
H12A—C12—H12C	109.5	O4—C26—H26C	109.5
H12B—C12—H12C	109.5	H26A—C26—H26C	109.5
O2—C13—H13A	109.5	H26B—C26—H26C	109.5
O2—C13—H13B	109.5	C4—N1—C1	109.95 (14)
H13A—C13—H13B	109.5	C4—N1—H11	124.0 (14)
O2—C13—H13C	109.5	C1—N1—H11	125.5 (14)
H13A—C13—H13C	109.5	C17—N3—C14	109.96 (12)
H13B—C13—H13C	109.5	C17—N3—H31	125.4 (12)
N3—C14—C15	109.19 (13)	C14—N3—H31	124.4 (12)
N3—C14—H14	125.4	C11—O1—C12	115.88 (14)
C15—C14—H14	125.4	C10—O2—C13	117.22 (14)
C14—C15—C16	105.46 (12)	C24—O3—C25	114.36 (12)
C14—C15—C19	124.65 (13)	C23—O4—C26	117.41 (11)

### Hydrogen-bond geometry (Å, º)

**Table:**

*D*—H···*A*	*D*—H	H···*A*	*D*···*A*	*D*—H···*A*
N1—H11···N2^i^	0.90 (1)	2.38 (2)	3.086 (2)	136 (2)
N1—H11···N2^ii^	0.90 (1)	2.55 (2)	3.250 (2)	136 (2)
N3—H31···O4^iii^	0.89 (1)	2.12 (1)	2.9327 (16)	151 (2)
N3—H31···O3^iii^	0.89 (1)	2.38 (2)	3.0709 (18)	134 (2)

Symmetry codes: (i) *x*, −*y*+5/2, *z*+1/2; (ii) −*x*+1, *y*+1/2, −*z*+1/2; (iii) *x*, −*y*+1/2, *z*+1/2.
